# Supplementary figures and images for: Modeling the MRI gradient system with a temporal convolutional network: Improved reconstruction by prediction of readout gradient errors
Source: Magn Reson Med. 2025 Aug 18;95(1):286–98. doi: 10.1002/mrm.70044 (PMC12620175; doi:10.1002/mrm.70044)

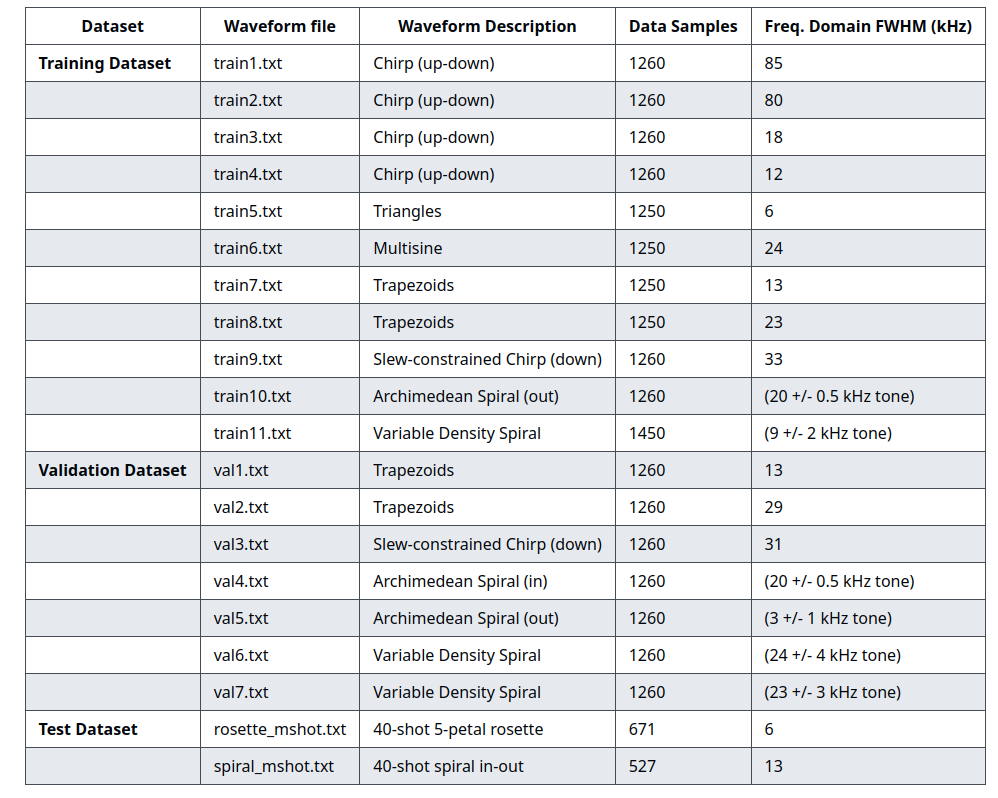

Supplement: Supplementary file 1 — Supporing Information [file MRM-95-286-s001.png]
